# Heterogeneity in the spread and control of infectious disease: consequences for the elimination of canine rabies

**DOI:** 10.1038/srep18232

**Published:** 2015-12-15

**Authors:** Elaine A. Ferguson, Katie Hampson, Sarah Cleaveland, Ramona Consunji, Raffy Deray, John Friar, Daniel T. Haydon, Joji Jimenez, Marlon Pancipane, Sunny E. Townsend

**Affiliations:** 1 Institute of Biodiversity, Animal Health and Comparative Medicine, College of Medical, Veterinary and Life Sciences, University of Glasgow, Glasgow, UK, G12 8QQ; 2 Animal Welfare Coalition, Dacon Building, 2281 Chino Roces Ave, Makati, Metro Manila, Philippines; 3 Department of Health, San Lazaro Compound, Santa Cruz, Manila, Metro Manila, Philippines; 4 Wise Monkey Foundation, 15600 NE 8th St, Suite B1, P #725, Bellevue, WA 98008, USA

## Abstract

Understanding the factors influencing vaccination campaign effectiveness is vital in designing efficient disease elimination programmes. We investigated the importance of spatial heterogeneity in vaccination coverage and human-mediated dog movements for the elimination of endemic canine rabies by mass dog vaccination in Region VI of the Philippines (Western Visayas). Household survey data was used to parameterise a spatially-explicit rabies transmission model with realistic dog movement and vaccination coverage scenarios, assuming a basic reproduction number for rabies drawn from the literature. This showed that heterogeneous vaccination reduces elimination prospects relative to homogeneous vaccination at the same overall level. Had the three vaccination campaigns completed in Region VI in 2010–2012 been homogeneous, they would have eliminated rabies with high probability. However, given the observed heterogeneity, three further campaigns may be required to achieve elimination with probability 0.95. We recommend that heterogeneity be reduced in future campaigns through targeted efforts in low coverage areas, even at the expense of reduced coverage in previously high coverage areas. Reported human-mediated dog movements did not reduce elimination probability, so expending limited resources on restricting dog movements is unnecessary in this endemic setting. Enhanced surveillance will be necessary post-elimination, however, given the reintroduction risk from long-distance dog movements.

Canine rabies is an important public health concern in developing countries, causing an estimated 55,000 human deaths in Africa and Asia every year and presenting a significant economic burden for both governments and families^1^. This continued rabies problem occurs despite successes in eliminating the disease from domestic dog populations in developed countries, and is the result of a long neglect of rabies control in low-income countries^2^. Evidence suggests that there are no insurmountable challenges to rabies elimination in Africa and Asia, with elimination by mass dog vaccination being both feasible and the most cost-effective means of human rabies prevention^3^,^4^,^5^. This efficiency of mass dog vaccination for rabies control has been demonstrated across continents and in a variety of local contexts^2^,^6^,^7^,^8^,^9^

The basic reproduction number of a disease, R_0_, describes the mean number of secondary cases produced by a single infected individual in a fully-susceptible population, and for rabies is consistently found to lie below two, typically around 1.2^8^,^9^. This low R_0_ means that vaccination coverage need only be maintained above around 20–45% to bring rabies under control, with eventual elimination^8^. In developing countries, however, high rates of dog population turnover rapidly erode population immunity, so that pulsed vaccination campaigns must reach higher coverage levels to maintain sufficient vaccinated dogs between campaigns^10^. Both empirical and theoretical evidence suggest that 70% coverage should be the target for annual vaccination campaigns^8^,^11^,^12^. However, while achieving high vaccination coverage is critical for success, this alone will not ensure elimination. Gaps in coverage reduce the probability of elimination by creating refuges where disease can remain in circulation^9^. Campaigns should, therefore, seek to achieve not just high coverage, but homogeneously high coverage.

The potential impacts of human-mediated transport of dogs on rabies elimination have rarely been investigated. A contact tracing study in Tanzania generated data on distances travelled by free-roaming rabid dogs^8^, but data on human-mediated movements are rare, limiting our understanding of their role in rabies epidemiology. Human-mediated animal movements have previously been found to be important for disease dynamics in both domestic animals^13^ and wildlife^14^. As dogs can be moved with relative ease, investigating such human-mediated movements may yield valuable insights into canine rabies dynamics. The little evidence available suggests that human-mediated dog movements are common in some areas, and can occur over considerable distances, occasionally spreading rabies between countries^15^,^16^,^17^,^18^. A recent modelling study suggested that human-mediated movements facilitated the rapid spread of rabies across the island of Bali^9^. This study also showed that the relative successes of different vaccination strategies for eliminating rabies depend on the frequency of human-mediated dog movement, and that, when vaccination coverage is spatially heterogeneous, frequent human-mediated movements reduce the probability of elimination. However, these results are specific to an epidemic situation, where rabies has been recently introduced to an area, and it is unclear whether they will hold in an endemic scenario, where rabies is already widespread.

The Philippines has suffered a high incidence of human rabies, and despite a long history of control efforts^19^, the disease remains endemic. Notwithstanding these past difficulties, mass dog vaccination could be very successful in the Philippines; domestic dogs are the sole rabies reservoir and elimination could be carried out island-by-island, with natural sea barriers limiting the risk of reintroductions^19^. Mass vaccinations are underway in Region VI of the Philippines (Western Visayas) as part of a rabies elimination demonstration project coordinated by the World Health Organisation (WHO) and funded by the Bill and Melinda Gates Foundation (BMGF). A household survey conducted in the region in early 2013 obtained information on dog ownership, human-mediated dog movements and dog vaccination status following the 2012 campaign. Here we used these data to determine the spatial pattern of vaccination coverage and estimate human-mediated dog movement rates in the region. This information was used to parameterise a spatially explicit, individual-based transmission model, previously developed to examine rabies elimination prospects^9^,^20^, to determine the likely consequences of the vaccination campaigns given alternative dog movement rates (including those estimated here for Region VI, and those previously inferred for Bali, Indonesia^9^). We used this model to explore the interplay between control efforts, both for endemic rabies as in Region VI, and for epidemic rabies as was the case for Bali. Finally, we investigate the efficacy of future vaccination scenarios. These analyses are a vital step towards ensuring programme targets are met through adaptive management, generating recommendations to improve prospects of rabies elimination within a reasonable timeframe.

## Results

### Vaccination coverage in Region VI

Vaccination coverage in Region VI at the time of the household survey was high in general, averaging 57% (95%CIs: 55.9–58.8%), though there was some variation at the province/city level (Table 1). Using the dog birth/death rate estimated from the household survey data, we back-calculated a regional coverage of 72% for seven months earlier, at the time directly after implementation of the 2012 campaign. Dividing numbers of dogs vaccinated in 2012 by the 2012 campaign coverage gave an estimated a total dog population of around 820,000 (95%CIs: 791,240–862,795) for Region VI. Campaign coverage estimates for Region VI in 2010 and 2011 (calculated by dividing dogs vaccinated in 2010 and 2011 by the dog population estimates) were also high at 73% and 78% respectively. In Bacolod City, coverage estimates during 2010–2011 were unrealistically high. Our approach may not accurately estimate population size in urban environments, where dog ownership patterns tend to be highly variable. However, Bacolod City represents only a small proportion of the total populated area.

While differences in province-level vaccination coverage were evident (Table 1), we also wanted to examine finer-scale heterogeneity. Region VI is divided into 133 municipalities/cities and 4,034 barangays (rural villages/city neighbourhoods), of which 20 municipalities and 100 barangays were included in the household survey. Vaccination coverage estimates at the barangay level were distributed roughly uniformly over 0–100% (supplementary Fig. S1), indicating substantial spatial heterogeneity. Of five barangay descriptors (and their two-way interactions) considered as fixed effects in a Generalised Linear Mixed Model (GLMM), only island (there are three main islands-Panay, Guimaras, and Negros-and any additional small islands were grouped with the nearest of these three) was retained in the best fitting model (Table 2). Mean vaccination coverage expected for barangays at the time of the survey was 45% for Panay, 67% for Guimaras, and 79% for Negros.

**Figure 1:**
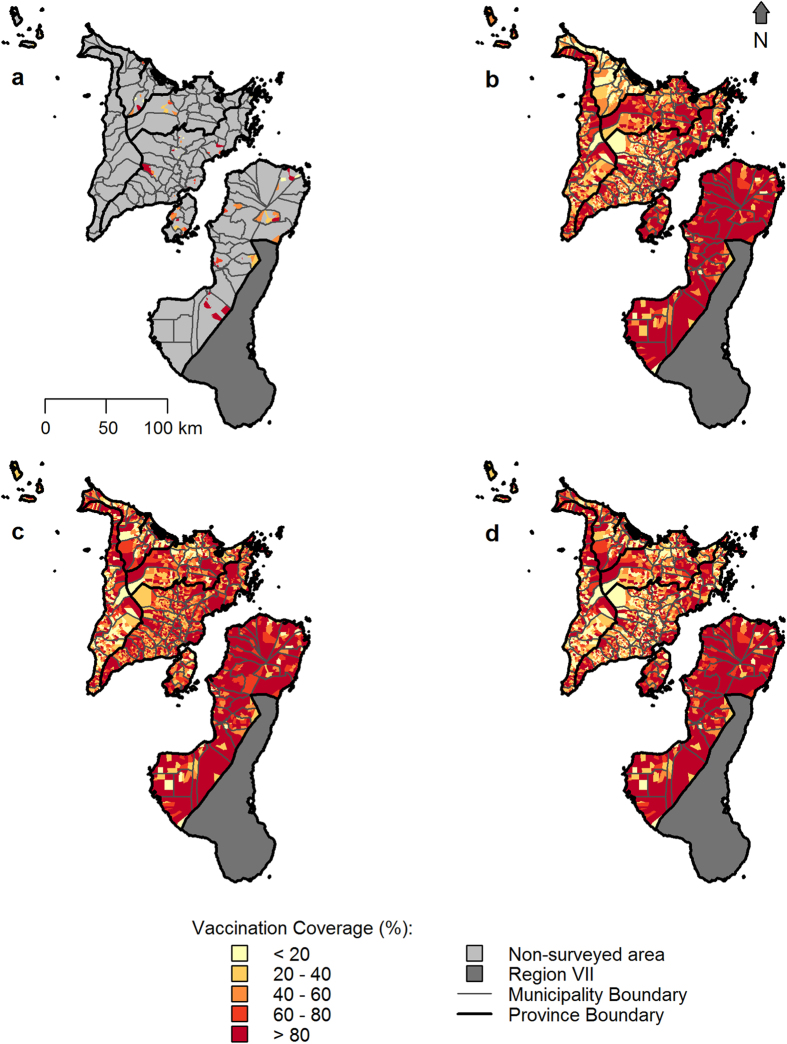
2010–2012 vaccination coverage in Region VI (**a**) Vaccination coverage at the time of the 2013 household survey for the 100 surveyed barangays. Extrapolated maps of vaccination coverage at the times of the campaigns in (**b**) 2010, (**c**) 2011 and (**d**) 2012. For non-surveyed barangays, 2012 coverage values were assigned based on island, municipality and random variation between barangays to achieve an appropriate level of heterogeneity. 2010 and 2011 values were obtained by adjusting 2012 values based on province. Maps were produced in R (version 2.15.2, R Core Team, 2012), using packages ‘maptools’ (version 0.8–30, Bivand, R. & Lewin-Koh, N., 2014), ‘maps’ (version 2.3–7, Becker, R. A., Wilks, A. R., Brownrigg, R. & Minka, T. P., 2014), ‘GISTools’ (version 0.7–3, Brunsdon, C. & Chen, H., 2014) and ‘RColorBrewer’ (version 1.0–5, Neuwirth, E., 2011).

The parameters estimated by the GLMM were used to develop barangay-level coverage maps of Region VI, which were used within our rabies transmission model. Coverage values were assigned to barangays that were not surveyed and projected back to the 2012 campaign, and to 2010 and 2011, to create a dynamic picture of vaccination in Region VI, incorporating realistic levels of heterogeneity (Fig. 1). While the estimated coverage of individual barangays changes on each iteration of this method due to the random assignment of 2012 coverage values, the overall statistical pattern remains consistent (supplementary Fig. S2). Thus, when using this method within the rabies transmission model (described below), we were able to represent the general pattern of vaccination in the region, while taking account of our uncertainty in the coverage of non-surveyed areas by creating a new coverage map on each model realization.

The most common reason given in the household survey for not vaccinating a dog was that the dog was too young (33.0% of households with unvaccinated dogs; Table 3), with other common responses being that dogs were difficult to handle (22.7%) and household members were unavailable on the vaccination day (14.7%). Reports that no vaccination was scheduled or information about vaccination was not received were also relatively common, at 12.1% and 10.7% of households with unvaccinated dogs respectively.

### Human-mediated dog movement rates

Rates of all types of human-mediated dog movement decline as the spatial scale of movement increases (Table 4). Within-municipality movement is common; we predict several hundred thousand such movements within the region annually (∼0.2% of the dog population daily). Movements between municipalities within the region’s provinces occur much less frequently (tens of thousands annually, or ∼0.02% of the population daily), and movements of Region VI dogs from their home provinces are comparatively rare (a few thousand annually, or ∼0.001% of the population daily). We estimate that around 650 dogs (95%CIs: 135–1,740) arrive in Region VI from outside the Philippines annually.

### Factors affecting vaccination campaign efficiency

The consequences of human-mediated dog movements and heterogeneous vaccination coverage for rabies elimination prospects in Region VI were assessed using a model of rabies transmission. Evidence from rabies-endemic areas suggests that typically less than 1% of the dog population is infected per annum^8^, so it is unlikely that susceptible depletion is the only mechanism limiting rabies transmission. As rabies incidence increases locally, people may more proactively confine or kill dogs that have been bitten or are showing signs of rabies^2^,^8^. An incidence-dependent human response of this kind could be influential in limiting transmission. This mechanism was introduced to the model via the constrained reproduction number R_C_, which describes the average number of cases caused by an infected dog in a high-incidence barangay (that experienced ≥5 cases in a month). R_C_ was set to less than R_0_ and likely lies below one, since values over one lead to unrealistically large outbreaks in poorly vaccinated barangays. Here we suggest 0.8 as a minimum value for R_C_, since this is the lowest value to which the reproduction number of rabies fell during efforts to control rabies in Bali, Indonesia^9^.

**Figure 2:**
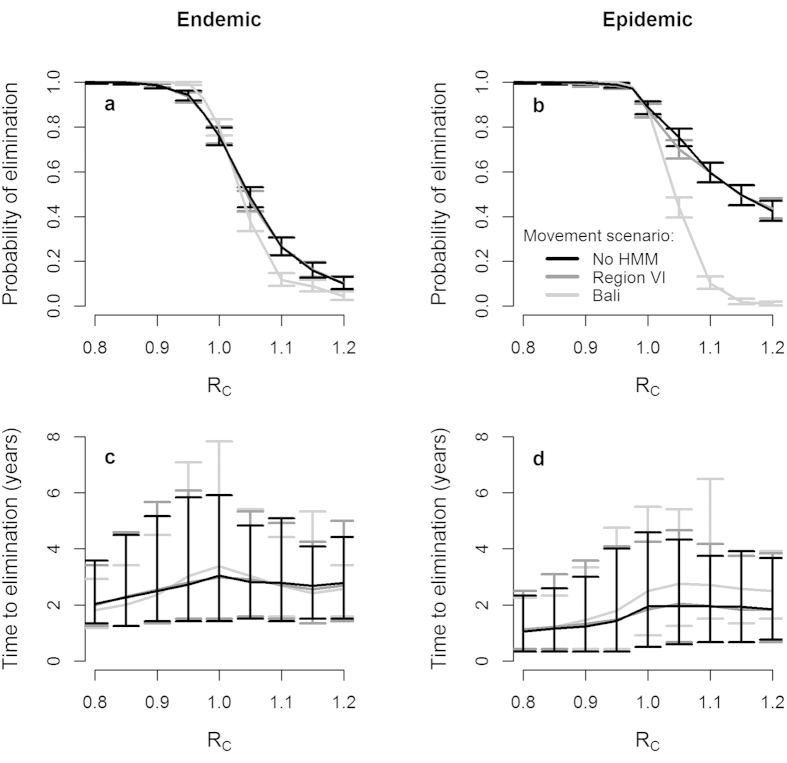
Consequences of human-mediated dog movements under endemic and epidemic conditions with varying incidence-dependent limits on local transmission. (**a,b**) Probability of rabies elimination from Region VI under (**a**) endemic and (**b**) epidemic scenarios, following three heterogeneous coverage vaccination campaigns based on those carried out annually during 2010–2012. Results were obtained for three human-mediated dog movement scenarios: no human-mediated movement (No HMM), and human-mediated movements as inferred for Region VI and for Bali^9^. Values from 0.8–1.2 were tested for the reproduction number (R_C_) to which rabies in a barangay was constrained following the occurrence ≥5 cases in a month. Binomial 95% confidence intervals are indicated (bars). (**c,d**) Time to elimination from the onset of vaccination for the simulations in (**a**) and (**b**). Means (lines) and 95 percentile intervals (bars) are based only on simulations where elimination was successful. 95 percentile intervals are obtained by removing the top and bottom 2.5% of observations of time to elimination from ordered lists of the values.

We ran simulations at values of R_C_ from 0.8 to 1.2 (R_0_), with a heterogeneous vaccination scenario consisting of three campaigns based on the 2010–2012 Region VI campaigns and three rates of human-mediated dog movement: (1) none; (2) spatially resolved rates estimated for Region VI (Table 4); and (3) a relatively high region-wide rate of 5%, as inferred for Bali, Indonesia^9^. We ran these simulations for an endemic scenario (like that in Region VI), where the disease was initially widespread, and an epidemic scenario, following a recent incursion. Elimination probability declined as R_C_ increased (Fig. 2a,b). The shape and extent of this decline, however, differed between movement scenarios. At R_C_≈1, elimination probability was roughly the same for all movement scenarios. As R_C_ increased above one, elimination probability was lowest for the highest movement scenario, with this effect being greater in an epidemic context. Rabies was easier to eliminate in the no movement and Region VI movement scenarios when disease was epidemic rather than endemic. As R_C_ fell below one, the elimination probability rose to one for all scenarios. This rise was most rapid for the highest movement scenario when disease was endemic, while there was little variation among scenarios for epidemic disease. Estimates of mean time to elimination (i.e. from the start of the initial vaccination campaign in 2010 until the final rabies case) consistently lay between one and 3.5 years across the R_C_ range for both epidemic and endemic scenarios, though there was a large amount of variability around these mean values (Fig. 2c,d).

**Figure 3:**
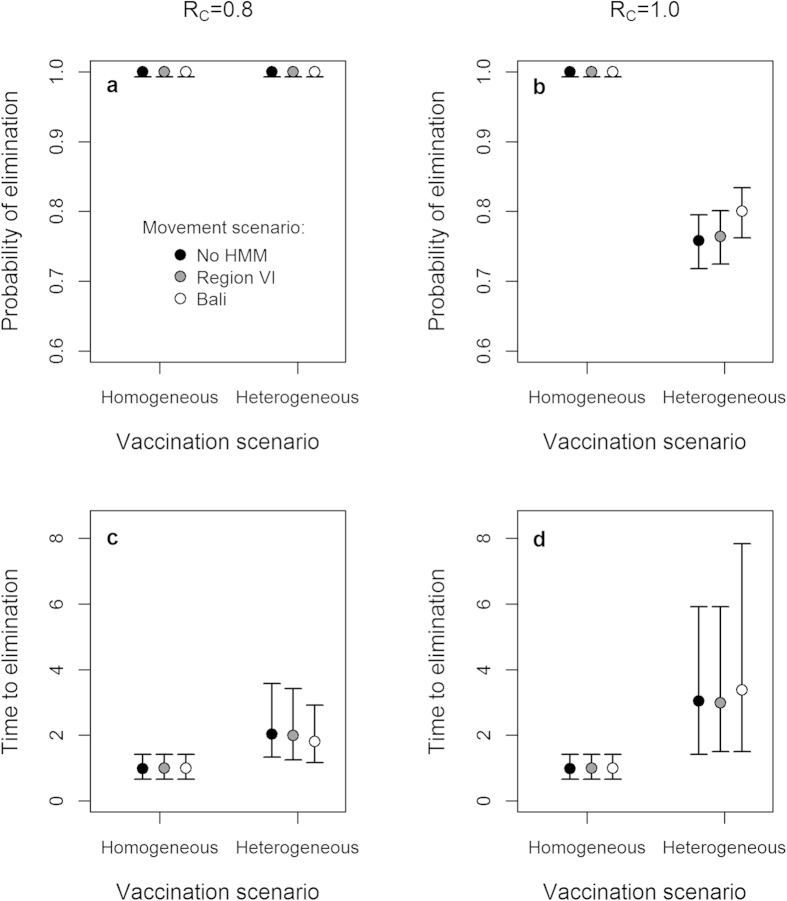
Consequences of heterogeneity in vaccination coverage. (**a,b**) Probability of rabies elimination from Region VI under homogeneous and heterogeneous vaccination coverage scenarios (involving 3 campaigns based on those carried out in 2010–2012) and three human-mediated dog movement scenarios: no human-mediated movement (No HMM), and human-mediated movements as inferred for Region VI and for Bali^9^. Results were obtained for situations where the reproduction number (R_C_) to which rabies in a barangay was constrained following the occurrence ≥5 cases within a month had a value of (**a**) 0.8 and (**b**) 1.0. Binomial 95% confidence intervals are indicated (bars). (**c,d**) Time to rabies elimination from the onset of vaccination for the simulations in (**a**) and (**b**). Means (points) and 95 percentile intervals (bars) are based only on simulations where elimination was successful.

We ran simulations for endemic rabies with the three dog movement rates under two vaccination scenarios: three heterogeneous campaigns based on those in Region VI in 2010–2012 (maps with observed levels of heterogeneity were produced such as those shown in Fig.1); and three campaigns with completely homogeneous coverage at the mean coverage values for the region in each year (Table 1). This analysis was undertaken at both R_C_ = 0.8 and R_C_ = 1.0, since the true value is expected to lie between these values. With R_C_ = 0.8, elimination occurred in 100% of simulations for all scenarios (Fig. 3a). Elimination was also guaranteed when R_C_ = 1.0 and vaccination was homogeneous. For R_C_ = 1.0 and heterogeneous vaccination, however, elimination probability was considerably reduced and varied slightly between movement scenarios (at R_C_ = 1.0 elimination probability is higher for the highest movement scenario, Fig. 2a). For both values of R_C_ (but especially R_C_ = 1.0), average time to elimination increased and showed more variability when coverage was heterogeneous (Fig. 3c,d).

Analyses of the sensitivity of the model to changes in the parameters governing the distributions of the number of offspring cases resulting from each rabies case and the generation interval (the time between a dog being exposed and transmitting rabies) show that, as we would expect, elimination prospects decline as the assumed values of R_0_ and the mean generation interval increase (supplementary Fig. S3). However, our findings that heterogeneous vaccination coverage gives reduced elimination prospects relative to homogeneous coverage and that human-mediated dog movements do not reduce elimination prospects (Fig. 2) are robust to changes in these parameters (supplementary Fig. S4).

### Goals for future campaigns

**Figure 4:**
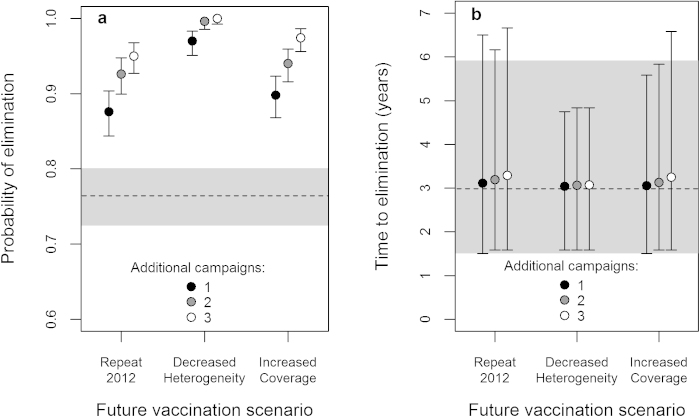
Comparison of rabies elimination prospects under future vaccination scenarios in Region VI. (**a**) Probability of rabies elimination in Region VI following an additional 1, 2 or 3 vaccination campaigns, which were either repeats of the coverage pattern achieved in the most recent campaign in 2012 (Repeat 2012), or versions of the 2012 campaign with either heterogeneity in coverage decreased by half (Decreased Heterogeneity), or coverage in each barangay increased by 10% (Increased Coverage). Binomial 95% confidence intervals are indicated (bars). The probability of elimination (dashed line) with 95% confidence intervals (shaded area) already achieved following the three campaigns during 2010–2012 is shown for comparison (dashed line). (**b**) Time from onset of mass vaccination in 2010 to rabies elimination for the same scenarios as (**a**). Means (points) and 95 percentile intervals (bars) are based on simulations where elimination was successful. The mean (dashed line) and 95 percentile interval (shaded area) following the 2010–2012 campaigns are shown for comparison.

We compared the effectiveness of three future vaccination scenarios in Region VI, in terms of rabies elimination prospects. For each scenario, we ran three sets of simulations differing in the total number of campaigns completed (four, five or six), with the first three campaigns for all scenarios based on those completed during 2010–2012. All scenarios were run at R_C_ = 1.0 to be conservative, since this is the highest this parameter is expected to be in reality. Results suggest that completing one additional campaign with the same coverage pattern as in 2012 would increase elimination probability from 0.76 (Fig. 3b) to 0.88 (Fig. 4a). We would expect completing two or three such additional campaigns to eliminate rabies with probabilities of 0.93 or 0.95 respectively (Fig. 4a). Implementing additional campaigns where the 2012 heterogeneity was halved (without increasing mean coverage), gave a higher elimination probability (0.97) after only one such campaign (Fig. 4a). Three future reduced heterogeneity campaigns led to elimination in all simulations. Applying a single future vaccination campaign where the coverage in each barangay was 10% higher than in 2012 gave an elimination probability intermediate to that achieved by a single repeat of the 2012 campaign and a single reduced heterogeneity campaign (Fig. 4a). Three increased coverage campaigns gave an elimination probability of 0.97.

For the scenarios where future campaigns had the same coverage pattern as in 2012 or achieved increased coverage, mean time to elimination increased slightly with the number of campaigns, since extending the campaigns over a longer time period allows additional successful eliminations to occur later. For the decreased heterogeneity vaccination scenario, however, mean time to elimination was consistent at three years, regardless of the number of additional campaigns (Fig. 4b). This is probably because, in 97% of cases (Fig. 4a), only one of these extra campaigns was sufficient to ensure rapid elimination; additional campaigns only aid in the last 3% of cases. Decreased heterogeneity campaigns gave less variability in time to elimination than the alternatives.

## Discussion

Understanding the factors influencing the effectiveness of vaccination programmes is vital in designing strategies for successful elimination of disease. We present a number of key results that increase our understanding of rabies elimination by mass dog vaccination and are relevant to the control and elimination of other infectious diseases. First, spatial heterogeneity in vaccination coverage can greatly reduce rabies elimination prospects, even when mean coverage is high. Reducing heterogeneity through a redistribution of vaccination effort could be more effective for disease elimination than an overall coverage increase. Second, we find that the negative effect of human-mediated dog movements on elimination prospects is greater when rabies has been recently introduced than in an endemic context. Third, local mechanisms that limit rabies spread (including euthanasia or confinement of rabid dogs) influence disease dynamics and potentially improve elimination prospects, reducing the negative impacts of coverage heterogeneity and human-mediated dog movement. For instance, human-mediated dog movements appear to be detrimental during an outbreak when there are only minimal social responses limiting transmission, but are less problematic (or even beneficial) when local interventions play a role.

Our results comparing homogeneous to heterogeneous vaccination coverage confirm previous modelling results that heterogeneity can be highly detrimental for the success of elimination programmes^9^. Additional empirical evidence for this comes from oral vaccination programmes in Europe to control rabies in foxes, where the number of campaigns required for elimination decreased as the comprehensiveness of vaccination coverage increased^21^. A previous study of rabies on the island of Bali, Indonesia, indicated that frequent human-mediated dog movements reduce the probability of elimination when vaccination coverage is heterogeneous^9^. While our results confirm this finding, we also show that, under endemic conditions such as in Region VI, the impacts of human-mediated movements are less pronounced. This is because of the widespread distribution of cases that will occur at the time of vaccination in an endemic context or following rapid dispersal of cases during an epidemic by frequent human-mediated movement. However, if long-distance transport of dogs is uncommon in an epidemic, the disease will remain relatively focal, and movement restrictions could improve elimination prospects by reducing the chance of dispersal to any poorly vaccinated areas.

Here, we also show that negative impacts of heterogeneity are mitigated by increased incidence-dependent limitations on local rabies spread, which result from human interventions such as confinement or euthanasia of dogs that are suspected to be infected^2^,^8^. As local transmission is limited, there is an increased likelihood of stochastic extinction from unvaccinated areas where disease could potentially persist. This effect, however, is likely scale-dependent; if coverage gaps are larger than those we explored at the barangay level, susceptible dog populations within these gaps may be sufficiently large to prevent stochastic extinction of rabies^22^. We assumed that there was no variation in the strength or spatial occurrence of mechanisms that curtail local transmission. However, we know very little about the extent and context specificity of social responses, which may be affected by rabies awareness or dog density. Research into these complexities, including the social and cultural responses and sensitivities to outbreaks of diseases such as rabies are an important area for future study, since they have a considerable bearing on the effectiveness of vaccination programs. Data on such interventions (e.g. numbers of suspected rabid dogs euthanised in communities, and the local incidence of rabies at the time when these euthanasias occurred) could be very informative. In the absence of such data for Region VI, we cannot be sure how large an effect the spatial heterogeneity in coverage had on control efforts. In the best-case scenario (R_C_ = 0.8), this heterogeneity may have only increased time to elimination by a year or so relative to homogeneous coverage. In the worst-case scenario (R_C_ = 1.0), elimination probability may have been reduced by 20–30%.

In both endemic and epidemic scenarios, negative effects of human-mediated movements decreased as incidence-dependent limitations on local transmission increased. This is probably because the increased probability of stochastic extinction means that, even when disease is introduced to a low-coverage barangay by long-distance movement, there is a greater chance that it will fail to establish and persist. Further work is required to confirm whether the observed interactions between dog movement and incidence-dependent limitations on local transmission are consistent under different vaccination strategies and human responses. Geographical characteristics also alter both human-mediated and natural dog movement. For example, rivers have been shown to impede the spread of both foot-and-mouth disease^23^ and rabies^14^, whilst coupled movement between urban centres drives the dynamics of many human diseases^24^,^25^. Model improvements could, therefore, incorporate such variations.

While simulations suggest that human-mediated dog movements have no negative impacts on vaccination campaign effectiveness in Region VI, these movements will pose a reintroduction risk once rabies has been eliminated and vaccination discontinued. We estimate that a few thousand dogs from Region VI are moved between Philippine provinces annually, with hundreds of additional dogs arriving in the region from other countries. Incursions have been a recurrent problem in Asia, for example in Bhutan^26^,^27^ and Indonesia^9^,^28^, with typically low levels of detection allowing the disease to establish^20^. It is therefore particularly important to maintain high levels of surveillance once elimination is achieved.

Had the vaccination campaigns in Region VI achieved homogeneous coverage, our results suggest that rabies would have rapidly been eliminated, probably prior to the implementation of the 2012 campaign. However, given the heterogeneity observed, in a worst-case scenario (R_C_ = 1.0), three further equivalent campaigns will likely be required to eliminate disease with high probability (0.95). More rapid elimination could reduce programme costs, which is pertinent given the limited available funding (for just two further campaigns). While it is unrealistic to expect any campaign to achieve completely homogeneous coverage, halving the heterogeneity observed in Region VI may be feasible. Our results suggest that this would be highly beneficial; one reduced heterogeneity campaign would increase elimination probability to >95%. A reduction in heterogeneity could be accomplished by actively targeting barangays missed during previous campaigns or where only low coverage was attained, even at the expense of reduced coverage in barangays where vaccination was previously very successful. Post-vaccination surveys to rapidly identify problematic areas in need of additional vaccination could be a worthwhile investment to target coverage improvements. Alternatively, the aim could be to increase overall coverage in future campaigns. Simulations suggest that this would be beneficial, though reducing heterogeneity would be more effective. For this reason, it may be best to focus efforts on identifying gaps and improving their coverage to reduce heterogeneity, with increasing overall coverage a secondary objective.

Survey responses as to why dogs in a household were not vaccinated provide suggestions for increasing coverage. Despite evidence that rabies vaccination provides effective pup protection^29^, the most common reason given for not vaccinating dogs was that they were too young, a problem evident in vaccination programmes elsewhere^30^,^31^. Better advertising of the need to vaccinate pups, and of the campaigns in general, should increase coverage. In addition, the high thermostability of some commercial rabies vaccines makes community-based approaches to pup vaccination between campaigns a feasible prospect to prevent build-up of susceptible individuals^32^. Carrying out more appropriately timed campaigns may also prove beneficial, as household members being busy on the vaccination day was another commonly reported issue. Simply ensuring that campaigns are implemented in all barangays is particularly important, since some households reported that no vaccination was scheduled locally. In addition, our finding that coverage on the island of Panay was generally lower than other areas in Region VI, suggests that increased effort should be focused there. In regions where there is no sustained programme of intensive vaccination in place, vaccination may decline at times of low rabies incidence^2^. However, newly implemented dog vaccination programmes, like that in Region VI, which aim to achieve high coverage in consecutive annual campaigns, can maintain this high coverage even when incidence declines by continued promotion of the need to vaccinate, and provision of free, local vaccination, which minimises costs to the owner.

We assumed an R_0_ of 1.2 for Region VI during our simulations, since this is typical of rabies in many countries^8^,^9^. Previous work suggests that rabies transmission is not dependent on the density of the dog population, so we did not allow R_0_ to vary spatially based on rural-urban status^8^,^9^. We also assumed no effect of unowned dogs on transmission dynamics, since evidence suggests that the proportion of dogs that are unowned is typically small in the Philippines and other parts of Southeast Asia^15^,^33^,^34^,^35^. Most dogs in this region, including owned dogs, are free-roaming^16^,^33^,^34^, but most are accessible for vaccination regardless of precise ownership status, allowing vaccination targets to be reached^36^. Furthermore, unowned dogs are unlikely to be involved in long-distance transmission of disease through human-mediated movement. Therefore, even if a small proportion of the dog population has not been captured by the household surveys, this is unlikely to affect elimination prospects.

Under realistic conditions for Region VI, we find no evidence that human-mediated dog movements are detrimental for rabies elimination prospects, suggesting that resources should not be spent on restricting dog movement. Long-distance dog movements will, however, present a reintroduction threat post-elimination, so effective surveillance and dog movement regulations will be critical at this stage. Our results suggest that annual vaccination campaigns in Region VI have been very successful, with all campaigns between 2010 and 2012 on average exceeding the 70% coverage recommended for achieving rabies elimination; an encouraging result for the feasibility of rabies elimination in Africa and Asia. Spatial heterogeneity in coverage, however, may be limiting the effectiveness of campaigns. We recommend that future campaigns aim to reduce this heterogeneity by increasing efforts in poorly vaccinated areas, even if this means reallocating resources from areas of high coverage.

## Methods

### The Household Survey

The municipalities/cities of Region VI were stratified based on four variables; rural/urban, coastal/inland, presence of a border with Region VII and island (Panay/Guimaras/Negros). Twenty municipalities/cities were then selected, randomly within strata. Five barangays were randomly selected from each of the 20 municipalities, and questionnaires (see Supplementary Information) completed within 30 households in each of these barangays (total sample size of 3,000 households). If fewer than 15 of the 30 households initially surveyed in a barangay did not own dogs, then the survey continued, targeting only dog-owning households, until 15 dog-owning households were reached, ensuring a minimum amount of data on dogs was collected per barangay. This household survey was implemented by the Department of Health in the Philippines as part of the WHO/BMGF rabies elimination project. The protocols were approved by the Philippines Departments of Health and of Agriculture, their respective field units in the study regions, provincial or city/municipal health and veterinary officers, mayors and barangay heads prior to the start of household surveys. The study was carried out in accordance with the protocols approved by these authorities. Informed verbal consent was obtained from all survey participants before carrying out the interviews.

Data were collected on paper questionnaires and then entered into tailor-made forms created using the Wise Monkey Portal, software developed in collaboration between the Wise Monkey Foundation (http://www.wisemonkeyfoundation.org/) and the University of Glasgow. The Portal hosts web applications and associated data centrally in the cloud, allowing immediate and secure access to registered personnel on any device connected to the internet. Data entry controls are used to maintain data quality, such as requiring certain fields, restricting freeform data entry, and providing a geographical hierarchy to record the location of activities. The Portal facilitated the timely analysis of quality data from the household survey, allowing rapid feedback of results to regional and national rabies program managers.

### Vaccination coverage in Region VI

A GLMM was used to identify predictors of barangay-level vaccination coverage at the time of the survey. The response variable described individual dog vaccination status, so the data were modelled as binomial variates. We considered five barangay descriptors as explanatory variables; island, province, urban/rural status, coastal/inland status, and presence of a border with Region VII. Municipality was incorporated as a random effect. Initial model fits produced residual deviances of an order of magnitude greater than the residual degrees of freedom, indicating substantial overdispersion. To overcome this, we modelled the excess variation using an observation-level (i.e. barangay-level) random effect, and this removed evidence of overdispersion. Starting with the most complex model, containing all two-way interactions between explanatory variables, terms were sequentially eliminated based on likelihood ratio tests, taking a test P-value of <0.05 to indicate significant support for the more complex of two nested models. Since province and island provide similar information (two of the three islands are represented by a single province), model selection was carried out twice, excluding the island effect and the province effect respectively. The resulting two simplified models were compared, and the best-fitting one selected as the final model.

Using the parameters estimated by the final GLMM, we built coverage maps of the region with realistic patterns of heterogeneity. This involved randomly assigning each non-surveyed barangay a vaccination coverage (*VC*) using the formula:


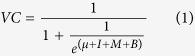


where *μ* was the intercept estimated by the GLMM; *I* was the fixed effect of the barangay’s island (the only fixed effect retained in the model); *M* was the random effect of the barangay’s municipality (drawn randomly for each municipality from 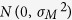, where 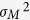 i-s the estimated municipality variance); and *B* was the random effect of barangay (drawn randomly from 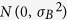), where 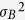 is the estimated barangay variance. Coverage values were then back-calculated to the time of the 2012 campaign (seven months before the household survey) by dividing by 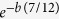, where *b* = birth/death rate (year^−1^), assuming constant population size. We set *b* to 0.38, the birth rate calculated from the household survey data, which included information on births per household in the last year. We also checked that none of the resulting coverage values exceeded 100%. To produce 2010 and 2011 barangay coverage values, we adjusted each of the 2012 coverage values by the change in mean coverage relative to 2012 in the corresponding province/city, for each year. The resulting 2010–2012 coverage estimates were mapped and used in the rabies transmission model described below.

### Human-mediated dog movement rates

Data were collected during the household survey on both permanent and temporary human-mediated dog movements at four spatial scales: between barangays within a municipality; between municipalities within a province; between provinces within the Philippines; and between the Philippines and other countries.

Permanent movement data were available as the reported locations from which household dogs were obtained, from which we calculated proportions of dogs moved at each scale. Since there was no date recorded for permanent movements (we only know that these movements occurred once in a dog’s lifetime), annual rates of permanent movement were estimated by dividing the proportions of dogs moving at each scale by two; the median age of dogs in Bohol in neighbouring Region VII^30^.

For temporary dog movements, information was available on how often (daily, weekly, monthly or yearly) households moved their dogs at each spatial scale. We assumed that only juvenile or adult dogs (>3 months) moved temporarily. Since households were not asked how many of their dogs were moved, upper and lower estimates of the temporary movement rates were obtained by assuming that all or only one of the adult dogs in a household were moved respectively. Annual temporary dog movement rates were determined by calculating total temporary movements made by surveyed adult dogs at each spatial scale in a year, dividing by the number of adult dogs surveyed, and multiplying by the proportion of dogs that were adults.

Total dog movement rates were obtained by summing temporary and permanent movement rates. Percentages of the dog population moving at each scale daily (used within the rabies transmission model outlined below) and total annual movements were obtained using the estimated dog population size.

### Modelling rabies elimination prospects

We employed an individual-based, spatially explicit rabies transmission model, which simulated rabies cases over a grid of 1 km^2^ cells representing Region VI. Cases were each allocated a generation interval from a gamma distribution (mean = 24 days, variance = 380 days)^8^ describing the time between exposure and transmission, and a grid cell, representing the location from which the case generated a number of offspring cases through transmission. The number of offspring cases resulting from each parent case was drawn from a negative binomial distribution with dispersion parameter 1.33 and mean equal to the effective reproduction number (R_e_) of rabies in the parent case’s grid cell^8^. R_e_ was calculated by multiplying R_0_ (set to 1.2) by the proportion of unvaccinated dogs in the cell.

Human-mediated movement was simulated by specifying an offspring case’s location relative to its parent as a random grid cell in another barangay within the municipality, another municipality within the province or another province within the region, each with a given probability. Alternatively, the location of an offspring case was assumed to arise from local, free-roaming dog movement with a probability of one minus the sum of the three human-mediated movement probabilities. For local movement, an offspring case’s location relative to its parent was determined by drawing a distance from a dispersal kernel (gamma-distributed: mean = 0.9 km, variance = 3.6 km)^8^ and applying a random movement direction. Since our main interest was in rabies elimination prospects, not the subsequent reintroduction risk, we assumed a closed system; one affected by dog births and deaths, but not by dogs entering or leaving the region.

We introduced a mechanism to the model that limited local rabies transmission in high incidence barangays, simulating incidence-dependent social responses to high local rabies incidence, such as restriction or euthanasia of bitten or rabid dogs. In grid cells in barangays that had experienced ≥5 cases in the last thirty days, we set R_e_ to the lower of either its usual value (R_0_ multiplied by the proportion of unvaccinated dogs) or a parameter R_C_; the constrained reproduction number, which had a value ≤R_0_. A lag period was also implemented, whereby this constraint continued for three months after ≥5 monthly cases were first recorded. After this period, the restriction was lifted or, if the barangay was still experiencing a high case frequency, it was extended for another three months. This feature simulated a situation where people in areas that have had recent outbreaks remain vigilant for some time. The choice of five monthly cases as the point at which to introduce the restriction was arbitrary, but deemed justifiable following comparison of the distribution of barangay outbreak sizes produced by the model with those obtained from data from Tanzania^8^ and Bali^9^.

We investigated two scenarios for initialising rabies cases in the model, the first being an endemic scenario, mimicking a situation, like in Region VI, where rabies is already widespread. In rabies-endemic areas, up to ∼1% of the dog population is expected to become rabid in a year^8^, or around 8,200 cases annually based on the Region VI population. Given a mean generation interval of 24 days, we expect 539 rabies cases per generation interval, and, therefore, initialised the model’s endemic scenario with 539 randomly scattered cases. The number of offspring cases per rabid dog was fixed to one (i.e. R_0_ = 1) for an initial six-month period, to generate a stable transmission pattern. A vaccination scenario was then applied and R_0_ was released to 1.2.

The second model initialisation scenario simulated a rabies epidemic resulting from a single randomly located point of introduction. Following the initial case, transmission was allowed to continue until 7,000 cases were reached, after which a vaccination scenario was applied (an estimated 7,000 cases occurred prior to the implementation of control efforts following the introduction of rabies to Bali, Indonesia^9^). Simulations where rabies did not establish (extinction before 7,000 cases) were discarded. If R_C_ was <1, we ran the first 7,000 cases using a value of one instead, before switching to the scenario-specific value. Imposing too strict a case limitation made establishment of an epidemic difficult, and may be unrealistic as awareness of how to respond to rabies is likely to be limited in the early stages of an epidemic.

Four vaccination scenarios were developed, all assuming that vaccination campaigns were delivered over three month periods, followed by gaps of nine months, with a third of municipalities being vaccinated during each campaign month. This structure resembled the implementation of campaigns in Region VI. All scenarios began with three campaigns based on those during 2010–2012. During campaigns in the ‘homogeneous’ scenario, every grid cell was assigned the mean coverage value achieved by that year’s campaign. In the ‘heterogeneous’ scenario, campaigns were simulated using the 2010–2012 barangay-level coverage maps developed above, so that each cell was assigned the coverage specified for its barangay. The 2010–2012 campaigns in the ‘reduced heterogeneity’ scenario were the same as in the ‘heterogeneous’ scenario, but any subsequent campaigns had their heterogeneity halved relative to 2012. This was achieved by calculating a new coverage value *n* for each barangay from the 2012 coverage *c* and the mean *µ* of the 2012 barangay coverage values, using the formula: *n* = ((*c−µ*)/2) + *µ*. In the ‘increased coverage’ scenario, the 2010–2012 campaigns were, again, the same as for the ‘heterogeneous’ scenario, but, in future campaigns, each barangay had its coverage increased by 10% (barangays which already had >90% coverage in 2012 were instead assigned a coverage of 100%). This also led to a small reduction in heterogeneity, since coverage then lay in the range 10–100%, rather than 0–100%.

Once a grid cell had been vaccinated, its coverage waned daily due to immunity loss and dog births and deaths at rate λ; calculated as 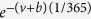, where *v* = 1/(duration of immunity in years) and *b* = birth/death rate (year^−1^). Duration of vaccine-induced immunity was set to two years based on Rabisin, the vaccine procured by the WHO.The commercial, high-quality vaccine used in the campaigns in the Philippines (Rabisin**®**, Merial) is known to be highly immunogenic with 97% of dogs in Europe still showing ‘protective’ antibody titres of >0.5 IU/ml when sampled 4 months after vaccination^37^.A higher proportion than this is actually likely to have been protected by vaccination, as antibody titres measured at 4 months after vaccination may already be starting to decline, even though dogs will still be protected. We therefore consider that genuine vaccine failures are likely to have been negligible. Incorrect storage or delivery of vaccine could have resulted in small numbers of ‘vaccinated’ dogs being unprotected, but the model conservatively assumes that vaccination coverage declines rapidly immediately after vaccination, which should capture any rare cases of failure of vaccine or vaccine delivery.

We developed three human-mediated dog movement scenarios to be used in combination with the vaccination scenarios. In the first of these, the probability of human-mediated movement was zero. For the second scenario, the probabilities of human-mediated movement at each spatial scale were selected, based on the daily percentages of the dog population moving (Table 4), to resemble movement patterns in Region VI. In the final movement scenario, rabid dogs had a 5% chance of moving to a random location, equivalent to the movement pattern inferred for Bali^9^. This last scenario involves much more long-distance human-mediated movement than was observed for Region VI (Table 4).

Simulations continued until rabies was successfully eliminated or until the monthly number of cases reached endemic levels at any time more than three years after the final campaign, indicating elimination failure. Model outputs reported whether rabies was eliminated and, if so, how long elimination took. All analyses described in the results were performed on 500 simulations of each scenario.

The rabies transmission model was developed in MATLAB (release 2013a, The MathWorks Inc.). Statistical analyses were completed in R (version 2.15.2, R Core Team, 2012), with Quantum GIS (version 1.8.0, Quantum GIS Development Team, 2013) also used for spatial analyses.

## Additional Information

**How to cite this article**: Ferguson, E. A. *et al.* Heterogeneity in the spread and control of infectious disease: consequences for the elimination of canine rabies. *Sci. Rep.* **5**, 18232; doi: 10.1038/srep18232 (2015).

## Supplementary Material

Supplementary Information

## Figures and Tables

**Table 1:** Vaccination coverage (VC) during the 2010–2012 campaigns in Region VI.

Province/City	Survey VC (%)	Campaign VC 2012 (%)	Dogs Vax 2012	Dog Population	Dogs Vax 2011	Campaign VC 2011 (%)	Dogs Vax 2010	Campaign VC 2010 (%)
**Aklan**	54 (49.0–59.4)	68 (61.4–74.4)	35,876	52,797 (48,236–58,420)	33,235	63 (56.9–68.9)	12,646	24 (21.6–26.2)
**Antique**	24 (17.9–32.4)	31 (22.4–40.6)	25,962	84,870 (63,899–116,030)	25,684	30 (22.1–40.2)	37,232	44 (32.1–58.3)
**Capiz**	48 (42.5–54.3)	61 (53.2–67.9)	49,478	81,694 (72,827–92,920)	54,401	67 (58.5–74.7)	64,422	79 (69.3–88.5)
**Guimaras**	61 (56.3–65.3)	76 (70.4–81.7)	13,518	17,740 (16,540–19,190)	11,328	64 (59.0–68.5)	15,500	87 (80.8–93.7)
**Iloilo**	49 (46.7–51.9)	62 (58.5–65.0)	202,091	327,180 (310,722–345,451)	280,975	86 (81.3–90.4)	224,100	69 (64.9–72.1)
**Negros Occidental**	68 (65.1–70.0)	85 (81.6–87.6)	170,778	201,748 (194,909–209,386)	167,088	83 (79.8–85.7)	187,766	93 (89.7–96.3)
**Iloilo City**	63 (57.1–69.2)	79 (71.5–86.6)	27,491	34,662 (31,735–38,465)	41,207	76 (68.3–82.7)	19,166	55 (49.8–60.4)
**Bacolod City**	68 (60.0–74.2)	85 (75.1–92.9)	16,617	19,663 (17,883–22,129)	26,253	100	40,110	100
**Region**–**wide**	57 (55.9–58.8)	72 (70.0–73.7)	541,811	820,354 (791,240–862,795)	640,171	78 (74.2–80.9)	600,942	73 (69.7–75.9)

Survey VC values were calculated from the 2013 household survey data, and 2012 campaign VC values were back-calculated from these to the time of the campaign. Dog populations were estimated by dividing dogs vaccinated in 2012 by 2012 campaign VC values. Dividing dogs vaccinated in 2011 and 2010 by these population estimates gave campaign VC values in these years. 95% confidence intervals are indicated in brackets.

**Table 2:** Output summary for barangay-level vaccination coverage GLMM.

Fixed Effects	Estimate	Standard Error	z-value
Intercept	0.708	0.749	0.946
Island (Negros)	0.606	0.852	0.946
Island (Panay)	−0.891	0.814	−1.094
**Random Effects**	**Variance**		
Municipality	0.634		
Barangay	2.286		

This model, with binomial errors, was selected as having the best fit to the data based on likelihood ratio tests (taking a test P-value of <0.05 to show significant support for the more complex of two nested models). Results were obtained from a sample of 100 barangays (5 from each of 20 municipalities). A random intercept was incorporated for each municipality, and also for each barangay/observation to overcome overdispersion. The factor level to which fixed effect values refer is specified in brackets.

**Table 3:** Reasons for not vaccinating dogs at the household level.

Reason for not vaccinating	% response
Dog too young	33.0%
Dog difficult to handle	21.7%
Was at farm/busy on vaccination day	14.7%
No vaccination scheduled	12.1%
Did not hear information about vaccination	10.7%
Fear	3.7%
Expensive	3.3%
Moved	2.0%
Too far to vaccination point	1.0%
Dog was pregnant	0.9%
Other	2.7%

Percentage of the 1,000 households with unvaccinated dogs that confirmed each reason as a factor in their failure to vaccinate.

**Table 4:** Human-mediated dog movement rates at four spatial scales in Region VI.

Movement Scale	Permanent movement rate	Temporary movement rate		Total movement rate		Total annual movements		% Population moving daily	
		Lower Estimate	Upper Estimate	Lower Estimate	Upper Estimate	Lower Estimate	Upper Estimate	Lower Estimate	Upper Estimate
Between barangays within a municipality	0.0314 (0.0264–0.0373)	0.5726 (0.2491–1.1189)	0.7196 (0.3455–1.4369)	0.6040 (0.2800–1.1481)	0.7509 (0.3758–1.4659)	495,466 (229,684–941,840)	616,040 (308,329–1,202,536)	0.1655 (0.0767–0.3145)	0.2057 (0.1030–0.4016)
Between municipalities within a province	0.0123 (0.0092–0.0164)	0.0186 (0.0037–0.0766)	0.0497 (0.0086–0.2300)	0.0309 (0.0161–0.0861)	0.0620 (0.0205–0.2403)	25,360 (13,176–70,636)	50,899 (16,836–197,105)	0.0085 (0.0044–0.0236)	0.0170 (0.0056–0.0658)
Between provinces within the Philippines	0.0043 (0.0028–0.0063	0.0002 (0.0000–0.0014)	0.0002 (0.0000–0.0014)	0.0046 (0.0030–0.0066)	0.0046 (0.0030–0.0066)	3736 (2,476–5,420)	3736 (2,476–5,420)	0.0012 (0.0008–0.0018)	0.0012 (0.0008–0.0018)
Between the Philippines and other countries	0.0008 (0.0002–0.0021)	0	0	0.0008 (0.0002–0.0021)	0.0008 (0.0002–0.0021)	657 (135–1,740)	657 (135–1,740)	0.00022 (0.00005–0.00058)	0.00022 (0.00005–0.00058

Rate unit is movements/dog/year. Total annual movements of Region VI dogs were estimated assuming a dog population size of 820,354. 95% BCa bootstrap confidence intervals (calculated from 10,000 bootstrap samples) are indicated in brackets.
