# Supplementary material for: Heterogeneity in the spread and control of infectious disease: consequences for the elimination of canine rabies
Source: Sci Rep. 2015 Dec 15;5:18232. doi: 10.1038/srep18232 (PMC4678884; doi:10.1038/srep18232)
Supplement: Supplementary Information [file srep18232-s1.pdf]

Supplementary Information

Heterogeneity in the spread and control of infectious disease: consequences for the elimination of canine rabies

Elaine A. Ferguson[1]*, Katie Hampson[1], Sarah Cleaveland[1], Ramona Consunji[2], Raffy Deray[3], John Friar[4], Daniel T. Haydon[1], Joji Jimenez[3], Marlon Pancipane[2], Sunny E. Townsend[1]

[1] Institute of Biodiversity, Animal Health and Comparative Medicine, College of Medical, Veterinary and Life Sciences, University of Glasgow, Glasgow, UK, G12 8QQ; [2]Animal Welfare Coalition, Dacon Building, 2281 Chino Roces Ave, Makati 1200, Philippines; [3]Department of Health, San Lazaro Compound, Santa Cruz, Manila, Philippines; [4]Wise Monkey Foundation, 15600 NE 8th St, Suite B1, P #725, Bellevue, WA 98008, USA

*e.ferguson.2@research.gla.ac.uk

Questionnaire

The following questionnaire was used during the 2013 Region VI household survey to obtain information on rabies vaccination and awareness, and dog demography and movements.

**1. Details of location**
Interviewer…………………………………..Date ………………   Province ………………. City/Municipality ……………………Barangay……………………..   Sitio……………………Name of Barangay Captain …………………………….

**2. Details of head of the family**
**Full name for head of the family**: ……………….…………………………………<br>**Education**<br>[ ] None [ ] Primary [ ] Secondary [ ] Diploma [ ] Graduate [ ] Other, specify …………<br>**Occupation**<br>[ ] Fisherman [ ] Farmer [ ] Employed [ ] Business [ ] Carpenter [ ] Driver<br>[ ] Housewife [ ] Retired [ ] Unemployed [ ] Teacher [ ] Other, specify …………………<br>**Religion**<br>[ ] Christian [ ] Muslim [ ] Iglesia ni Cristo [ ] Baptist [ ] Other, specify……………………

**3. Details of household**
**No. of people aged 18 years or older** ……………….<br>**No. of people younger than 18 years old** …………………..<br>**Type of house (*tick all that apply*)**<br>[ ] bamboo [ ] wood [ ] bricks [ ] cement [ ] other, specify……………………..<br>**Are any dogs or puppies kept?**<br>[ ] YES [ ] NO **If NO, skip section 4.**

**4. Details of dogs**
**No. of dogs** ……………..<br>**No. of dogs vaccinated**……………<br>**No. of dogs unvaccinated**………………
**No. of dogs younger than 3 months**: ………….<br>**No. of dogs younger than 3 months and vaccinated**: …………………<br>**No. of certificates seen for vaccinated dogs younger than 3 months**:………………………<br>**No. of dogs age 3 months or older**: ………<br>**No. of dogs age 3 months or older and vaccinated**: …………………<br>**No. of certificates seen for vaccinated dogs age 3 months or older**:…………………
**Where dogs were vaccinated (*tick all that apply*):**<br>[ ] Barangay hall [ ] Animal clinic [ ] During house to house dog vaccination<br>[ ] Other, please specify …………………….
**Why dogs are not vaccinated (*tick all that apply*)**<br>[ ] too young [ ] moved [ ] difficult to handle [ ] was at farm/busy<br>[ ] not heard information [ ] fear [ ] expensive [ ] other, specify……………………..
**Why dogs are kept (*tick all that apply*)**<br>[ ] guard home [ ] guard livestock [ ] pets [ ] other, specify ……………………..
**Where dogs were born. No. dogs born in:**<br>household: ....<br>another place in barangay: ....<br>another barangay in the city/municipality: ....

another city/municipality in the province: ....
another province in the Philippines: ....
outside the Philippines: ....
If place of birth unknown, please specify reason:
[ ] bought at market [ ] adopted from shelter/pound [ ] adopted from elsewhere
[ ] lost when found [ ] gift [ ] other, specify ....

No. of puppies born in the household in past 1 year:..
No. of dogs younger than 3 months died in past 1 year:………………………
No. of vaccinated dogs younger than 3 months died in past 1 year:..................................
No. of dogs age 3 months or older died in past 1 year:..
No. of vaccinated dogs age 3 months or older died in past 1 year:.....................................

If your household had a litter of puppies in the past 1 year, where are those dogs now? No. of dogs:
Still in the household…………
another place in barangay: ....
another barangay in the city/municipality: ....
another city/municipality in the province: ....
another province in the Philippines: ....
outside the Philippines: ....

How often do you take your dog(s) outside the Philippines?
[ ] Never [ ] yearly [ ] monthly [ ] weekly [ ] daily
How often do you take your dog(s) to another province in the Philippines?
[ ] Never [ ] yearly [ ] monthly [ ] weekly [ ] daily
How often do you take your dog(s) to another city/municipality in this province?
[ ] Never [ ] yearly [ ] monthly [ ] weekly [ ] daily
How often do you take your dog(s) to another barangay in the city/municipality?
[ ] Never [ ] yearly [ ] monthly [ ] weekly [ ] daily

5. Awareness

Do you know that rabies can kill dogs and humans? [ ] Yes [ ] No
Do you know that rabies can be prevented by rabies vaccination of dogs and cats?
[ ] Yes [ ] No
If yes, where did you get such information?
[ ] TV [ ] Radio [ ] Print media [ ] Government personnel [ ] School
[ ] Community [ ] Other, please specify…………………………

Interviewer comments

Figures

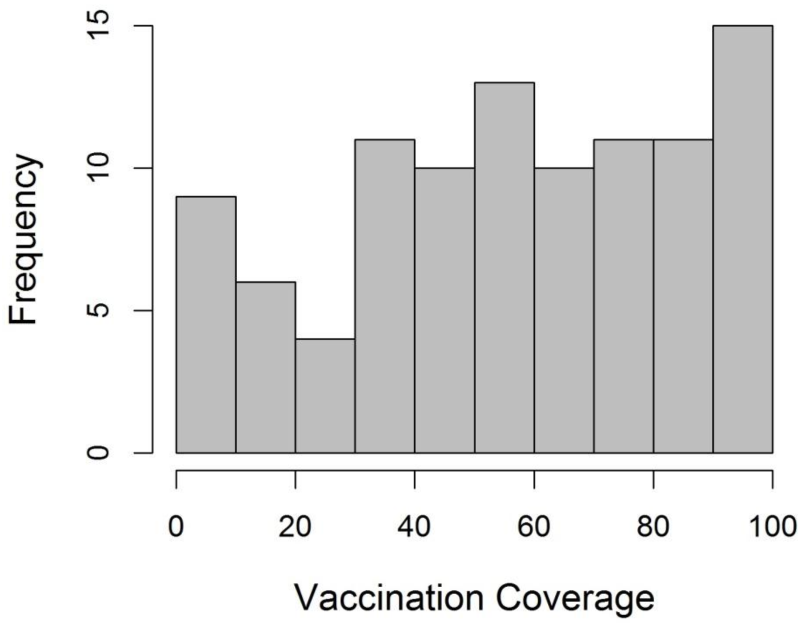

Figure S1: Histogram of barangay-level vaccination coverage at the time of the 2013 household survey. Based on a sample of 100 barangays from 20 municipalities.

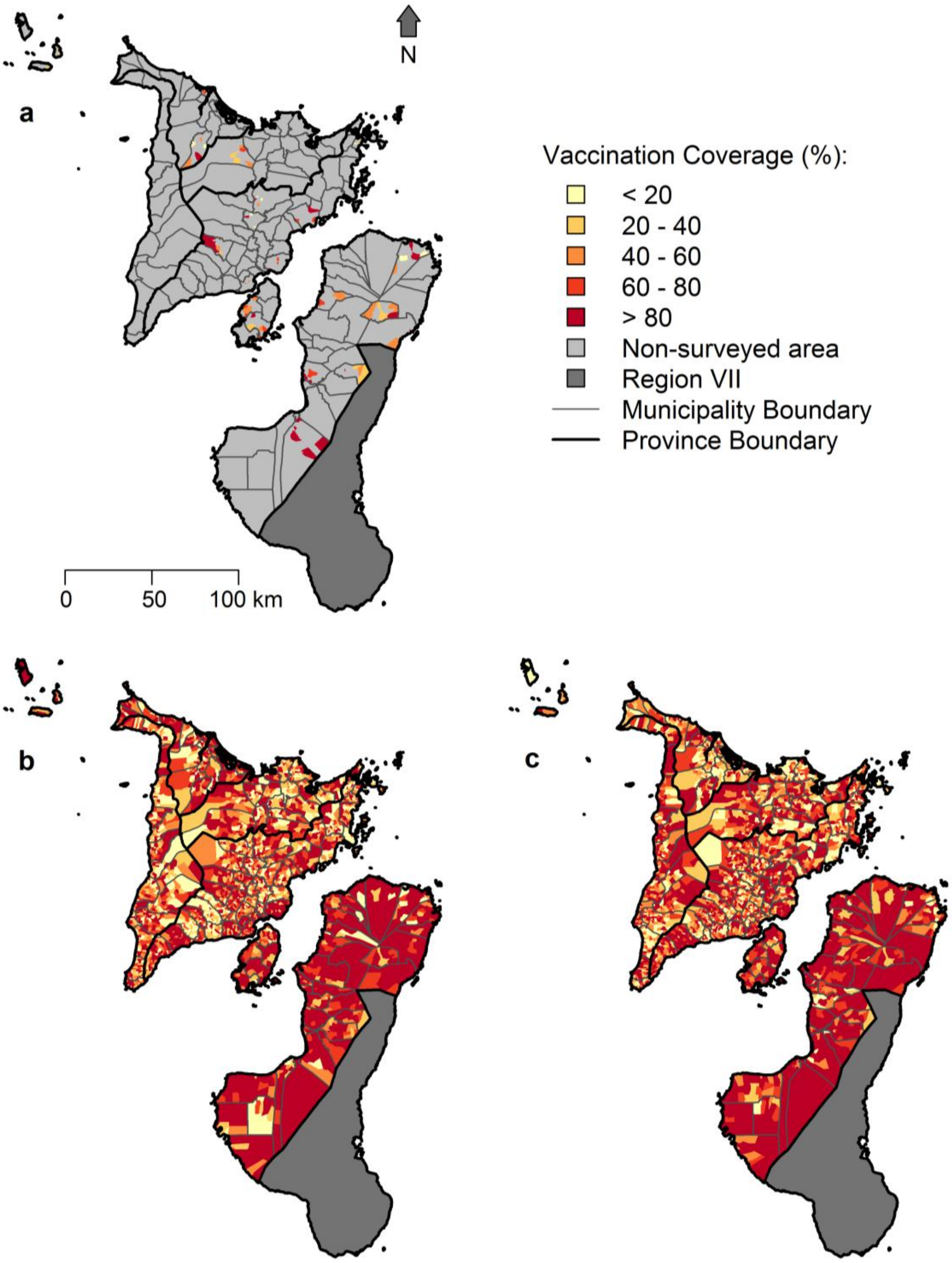

Figure S2: Extrapolating the 2012 barangay-level vaccination coverage pattern for Region VI. (a) Vaccination coverage in the 100 surveyed barangays at the time of the 2013 household survey. (b), (c) Examples of the vaccination coverage pattern extrapolated for the 2012 campaign. For non-surveyed barangays, values were assigned taking account of island, municipality and random variation between barangays. Maps were produced in R (version 2.15.2, R Core Team, 2012), using the packages 'maptools' (version 0.8-30, Bivand, R. & Lewin-Koh, N., 2014), 'maps' (version 2.3-7, Becker, R. A., Wilks, A. R., Brownrigg, R. & Minka, T. P., 2014), 'GISTools' (version 0.7-3, Brunsdon, C. & Chen, H., 2014) and 'RColorBrewer' (version 1.0-5, Neuwirth, E., 2011).

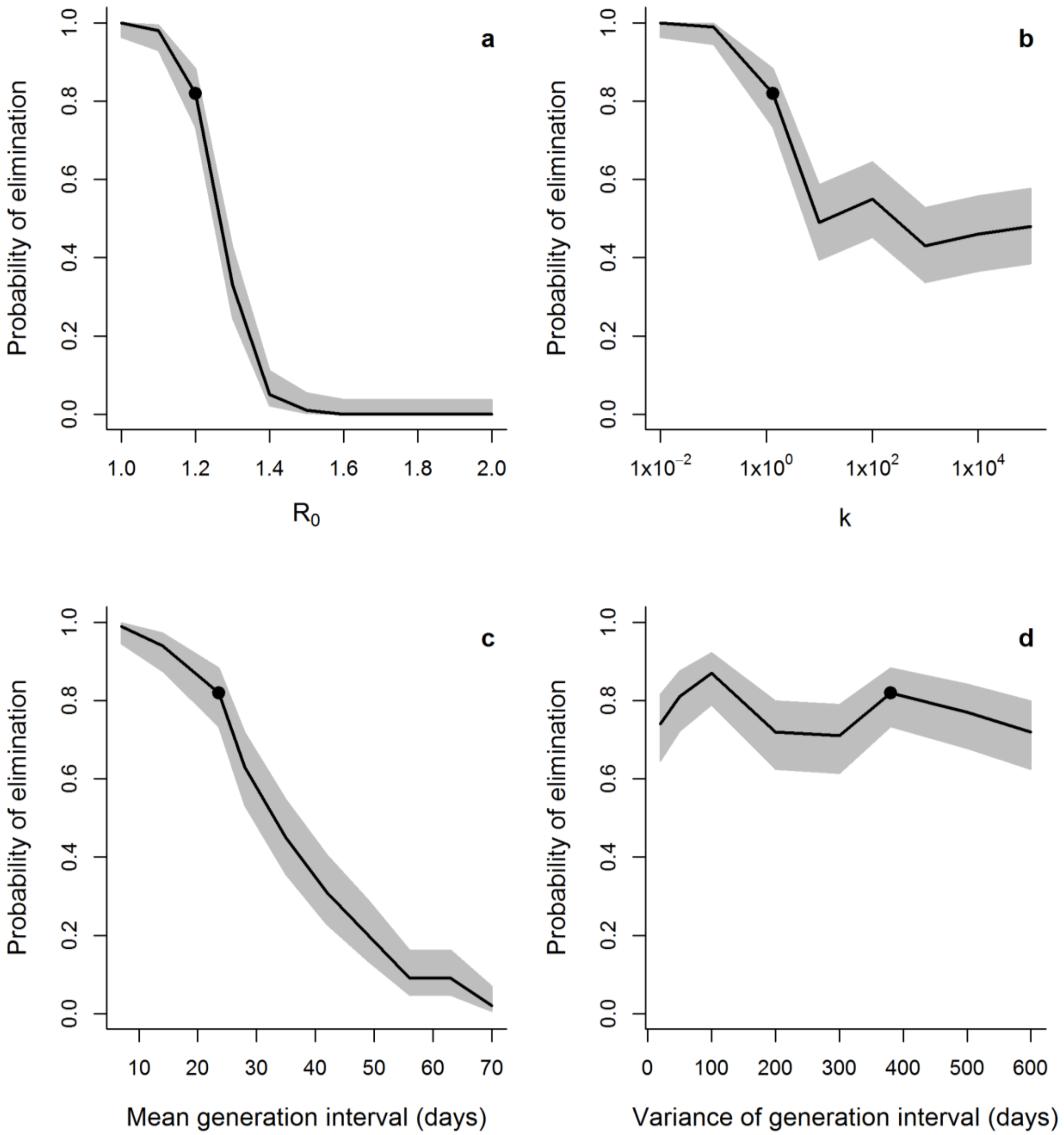

Figure S3: Sensitivity to model parameters. An assessment of the sensitivity of our results regarding the probability of elimination of endemic rabies from Region VI following the three heterogeneous vaccination campaigns from 2010-2012 (assuming no further campaigns) to the values of (a) R_0, (b) the dispersion parameter k of the negative binomial distribution describing numbers of offspring cases, and the (c) mean and (d) variance of the gamma distribution describing the generation interval. Results were generated assuming levels of human-mediated dog movement inferred for the region from household survey data, and an R_C of 1.0. 100 model simulations were used to calculate each elimination probability. Points indicate the value assumed for each parameter to produce the results presented in the main text. Shaded areas indicate binomial 95% confidence intervals.

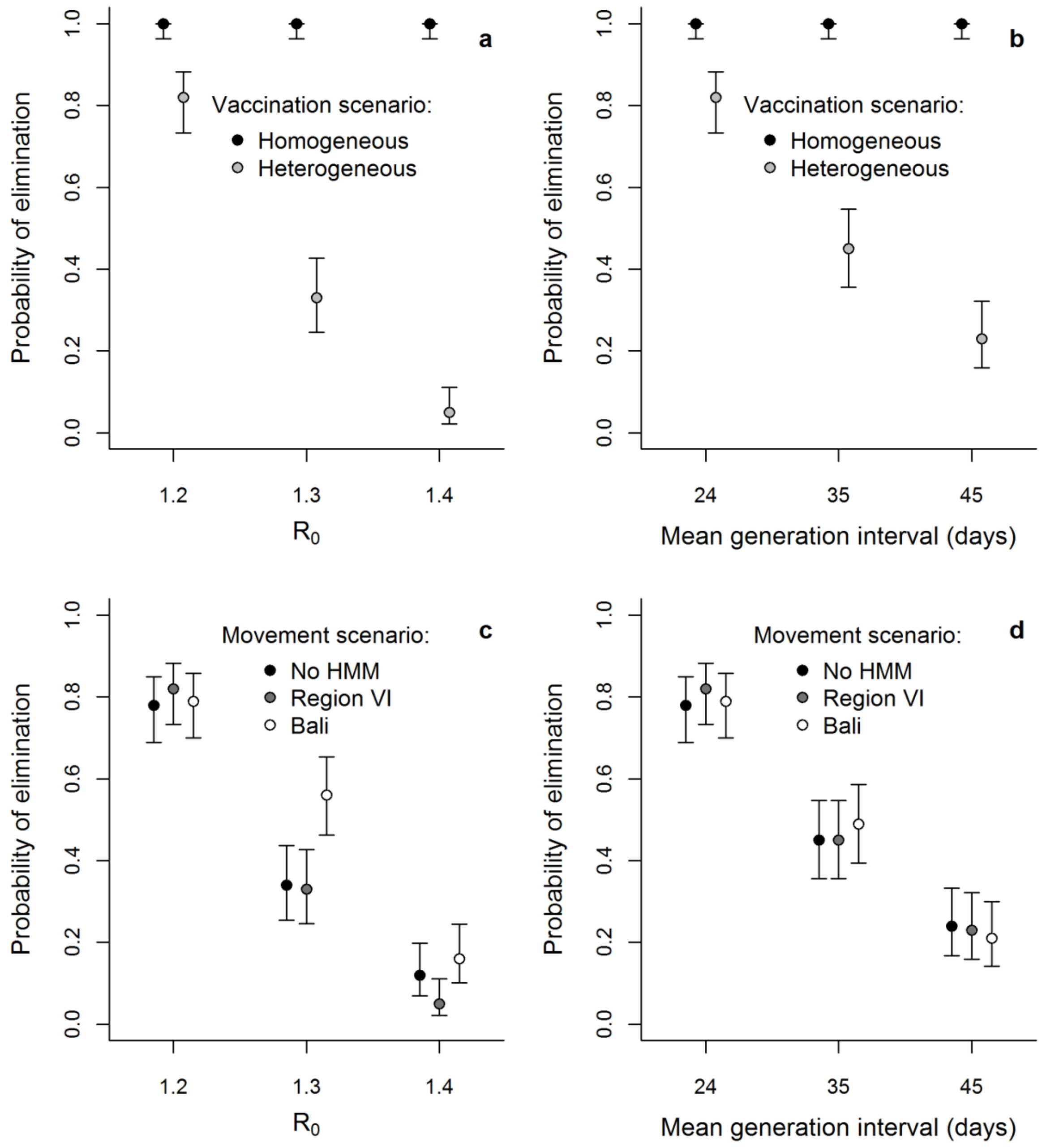

Figure S4: Robustness of key results. (a, b) Probability of rabies elimination from Region VI under homogeneous and heterogeneous vaccination coverage scenarios (involving 3 campaigns based on those carried out in 2010-2012) at three values of (a) R_0 and (b) the mean generation interval (including the values assumed to produce the results in the main text; 1.2 and 24 days respectively). (c,d) Probability of rabies elimination from Region VI following the three heterogeneous vaccination campaigns from 2010-2012, under three human-mediated dog movement scenarios (no human-mediated movement (No HMM), and human-mediated movements as inferred for Region VI and for Bali[9]) at three values of (c) R_0 and (d) the mean generation interval. All results were generated assuming an R_C of 1.0. Each point is based on 100 model simulations. Binomial 95% confidence intervals are indicated (bars).
